# *Trypanosoma cruzi* activates mouse cardiac fibroblasts in vitro leading to fibroblast-myofibroblast transition and increase in expression of extracellular matrix proteins

**DOI:** 10.1186/s13071-018-2614-1

**Published:** 2018-01-30

**Authors:** Laura Lacerda Coelho, Isabela Resende Pereira, Mirian Claudia de Souza Pereira, Liliane Mesquita, Joseli Lannes-Vieira, Daniel Adesse, Luciana Ribeiro Garzoni

**Affiliations:** 1 Laboratório de Inovações em Terapias, Ensino e Bioprodutos, Instituto Oswaldo Cruz, Fundação Oswaldo Cruz (Fiocruz), Av. Brasil 4365, Pavilhão Cardoso Fontes, 2° andar, Rio de Janeiro RJ, 20045–900 Brazil; 2 Laboratório de Biologia das Interações, Instituto Oswaldo Cruz, Fundação Oswaldo Cruz (Fiocruz), Av. Brasil 4365, Pavilhão Cardoso Fontes, 2° andar, Rio de Janeiro RJ, 20045–900 Brazil; 3 Laboratório de Ultraestrutura Celular, Instituto Oswaldo Cruz, Fundação Oswaldo Cruz (Fiocruz), Av. Brasil 4365, Pavilhão Carlos Chagas sala 308, Rio de Janeiro RJ, 20045–900 Brazil; 4 Laboratório de Biologia Estrutural, Instituto Oswaldo Cruz, Fundação Oswaldo Cruz (Fiocruz), Av. Brasil 4365, Pavilhão Carlos Chagas, sala 307, Rio de Janeiro RJ, 20045–900 Brazil

**Keywords:** Chagas disease, *Trypanosoma cruzi*, Fibrosis, Cardiac fibroblasts, Extracellular matrix

## Abstract

**Background:**

Cardiac fibrosis is a consequence of chronic chagasic cardiomyopathy (CCC). In other cardiovascular diseases, the protagonist role of fibroblasts in cardiac fibrosis is well established. However, the role of cardiac fibroblasts (CFs) in fibrosis during the CCC is not clear. Here, our aim was to investigate the effect of *Trypanosoma cruzi*, the etiological agent of Chagas disease on CFs activation.

**Methods:**

Cardiac fibroblasts were purified from primary cultures of mouse embryo cardiac cells. After two passages, cells were infected with *T. cruzi* (Y strain) and analyzed at different times for determination of infectivity, activation and production of extracellular matrix components (fibronectin, laminin and collagen IV) by immunofluorescence and western blot.

**Results:**

At second passage, cultures were enriched in CFs (95% of fibroblasts and 5% of cardiomyocytes), as revealed by presence of alpha-smooth muscle actin (α-SMA) and discoidin domain receptor 2 (DDR2) and absence of sarcomeric tropomyosin (ST) protein expression. *Trypanosoma cruzi* infection induced fibroblast-myofibroblast transition, with increased expression of α-SMA after 6 and 24 h post-infection (hpi). Fibronectin was increased at 6, 24 and 48 hpi, laminin was increased at 6 and 24 hpi and collagen IV was increased at 6 hpi.

**Conclusions:**

Our results showed that *T. cruzi* activates CFs, inducing activation and exacerbates ECM production. Furthermore, our data raise the possibility of the involvement of CFs in heart fibrosis during Chagas disease.

## Background

Chagas disease (CD) caused by the protozoan *Trypanosoma cruzi* is endemic in Latin America, affects about 7 million people around the world [1] and is one of the most important causes of infectious cardiomyopathy [2]. Thirty percent of infected people will develop the clinical symptoms of chronic CD, which include cardiac, digestive, cardio-digestive and neurological manifestations [3, 4]. The cardiac form of CD is the most significant clinical manifestation due to its gravity and frequency [3].

In chronic chagasic cardiomyopathy (CCC), the parasite persistence contributes to chronic inflammation and death of cardiac cells, resulting in fibrosis and heart failure [3–5]. In experimental CD, intracellular forms of *T. cruzi* and inflammatory infiltrates can be observed in association with fibrotic areas in the cardiac tissue [6, 7]. There are several previous studies that have suggested an increased expression of fibronectin, laminin and collagen IV expression upon *T. cruzi* infection [8] that was shown to be reversible after treatment with the trypanocidal agent posaconazole (L. R. Garzoni, personal communication). Such observations indicate that the infection per se, independently of the presence of inflammatory cells, contributes to tissue fibrosis. Moreover *T. cruzi* expresses a family of glycoproteins that bind to fibronectin and laminin. Upon adhesion to either of these proteins, a series of serine-, threonine- and tyrosine-kinases are activated that, in turn, phosphorylate flagellar tubulin from the parasite [9]. Conversely, the parasite’s cysteine proteinases degrade fibronectin for successful invasion of the host cell [10].

Fibrosis can be induced by distinct stimuli such as infections, toxins, drugs, tissue injury and persistent inflammation. When exacerbated, fibrosis can cause organ dysfunction and death [11]. Fibrosis is defined as excessive deposition of ECM proteins in organs and tissues, as a result of fibroblasts activation [12]. When activated, mainly in response to cytokines and growth factors such as TNF and TGF-β, fibroblasts transition to myofibroblasts and start expressing smooth muscle α-actin (α-SMA) and produce high amounts of ECM proteins [11, 13]. Fibroblasts are mesenchymal cells found in the connective tissue in distinct organs [14] and the role of these cells on genesis of fibrosis is well established [15].

Fibroblasts are the most abundant cell type found in the heart tissue, along with myocytes, endothelial and smooth muscle cells [16]. Cardiac fibroblasts (CFs) produce metalloproteases and their tissue inhibitors (TIMPs), growth factors, cytokines (including TNF, IL-1 and IL-6) as well as reactive oxygen and nitrogen species, contributing to the structure and function of the heart tissue [14, 17].

The involvement of CFs in fibrosis formation associated to hypertrophy in cardiovascular diseases including myocardial infarct, hypertension and heart failure is well established [14, 18]. However, the role of CFs in the genesis of cardiac fibrosis during CD has not been addressed. In this context, the aim of the present study was to investigate the effects of *T. cruzi* on activation of CFs trying to shed light on the involvement of these cells in the formation of cardiac fibrosis during CD. For this purpose, we established a purified culture of CFs that was successfully infected with *T. cruzi* and observed fibroblast-myofibroblast transition in infected cells. *Trypanosoma cruzi* also increased the expression of ECM proteins in the course of the infection, indicating that the direct infection of CFs in vivo, especially in the acute phase, may participate in the initiation of a fibrotic event, contributing to the pathogenesis of the chagasic cardiomyopathy.

## Methods

### Reagents and antibodies

Trypsin and ethylenediaminetetracetic acid (EDTA) were acquired from Gibco (Carlsbad, CA, USA). Type II collagenase was obtained from Worthington Biochemical Corporation (Lakewood, NJ, USA). Phosphate buffered saline (PBS), fetal bovine serum (FBS), L-glutamine, penicillin, streptomycin, CaCl_2_, Dulbecco’s Modified Eagle’s Medium (DMEM), RPMI, acetone and bovine serum albumin (BSA), were obtained from Sigma-Aldrich (St. Louis, MO, USA). Giemsa solution was obtained from Merck (Frankfurt, Germany). Primary antibodies, rabbit polyclonal antibodies anti-fibronectin and anti-laminin, mouse monoclonal antibody anti-α-SMA and mouse anti-ST were obtained from Sigma-Aldrich. Goat polyclonal antibody anti-DDR2 was obtained from Santa Cruz Biotechnology (Santa Cruz, CA, USA) and mouse monoclonal antibody anti-GAPDH was acquired from Fitzgerald (Acton, MA, USA). Secondary antibodies, goat anti-mouse IgG Alexa Fluor 594 or 488, goat anti-rabbit IgG and goat anti-mouse IgG HRP-labeled were obtained from Invitrogen (Carlsbad, CA, USA). BCA protein assay reagent (bicinchoninic acid) and 4′-6-Diamidino-2-phenylindole (DAPI) were acquired from Thermo Fisher Scientific (Rockford, IL, USA). 1,4-diazabicyclo [2.2.2] octane (DABCO) was obtained from Sigma-Aldrich. The Protease Inhibitor Cocktail was purchased from Roche Molecular Biochemicals (Indianapolis, IN, USA) and the chemiluminescent kit ECL from Pierce (Rockford, IL, USA).

### Isolation and purification of mouse cardiac fibroblasts

Heart ventricles were obtained from 18 day-old fetuses of Swiss Webster mice. Cells were isolated by mechanical and enzymatic dissociation methods using 0.05% trypsin and 0.01% collagenase in PBS (pH 7.2) at 37 °C, as previously described [19]. Cells were plated on 0.02% gelatin-coated 25 mm^2^ flasks and maintained at 37 °C in 5% CO_2_ atmosphere in DMEM supplemented with 10% FBS, 2.5 mM CaCl_2_, 1 mM L-glutamine, 2% chick embryo extract, 1000 U/ml penicillin and streptomycin 50 μg/ml. CF-enriched cultures were established from 3-day old primary cardiac cultures by dissociation with trypsin/ EDTA in HBSS without calcium and magnesium. The isolated cells were plated at a density of 10^6^ cells in 25 mm^2^ cell-culture flasks. Fully confluent cultures were split every 3–4 days and cells were used for experiments at second passage at the density of 5 × 10^5^ cells/dish for 60 mm culture dishes or 5 × 10^4^ cells/well in 13 mm round glass coverslips in 24-well plates. CFs were maintained at 37 °C in 5% CO_2_ atmosphere in DMEM supplemented with 10% FBS and 1000 U/ml penicillin and streptomycin 50 μg/ml (CF Medium).

### Infection with *Trypanosoma cruzi* (Y strain)

Trypomastigotes forms of the Y strain (MHOM/BR/1950/Y) were obtained from previously infected cultures of Vero cell. After 4 days of infection, the parasites released in the supernatant were harvested and centrifuged at 800 g for 20 min at 4 °C. CFs were infected after 24 h of plating at a multiplicity of infection of 10:1 (parasites: host cell) in 500 μl of DMEM without FBS. After 24 h of interaction, extracellular parasites were washed out and fresh medium with 10% FBS was added in case of long-term infection.

### Giemsa staining

CFs infection was interrupted at desired time-points (6 to 96 h) with Bouin’s fixative solution (picric acid-formalin-acetic acid mixture). The samples were dehydrated in acetone/xylene gradient and stained in Giemsa solution. Coverslips were mounted with Permount resin and the images were acquired using bright field microscopy (Nikon Eclipse E200, Nikon, Tokyo, Japan) and analyzed with the software Nis-Elements BR 4.0.

### Immunofluorescence

CF cultures were washed with PBS and fixed for 5 min at 20 °C with 4% paraformaldehyde in PBS. After washing, cells were permeabilized with 0.5% Triton X-100 and non-specific antibody binding was blocked with PBS containing 4% BSA. Then, the cells were incubated overnight at 4 °C with primary antibodies including mouse anti α-SMA, mouse anti-ST, rabbit anti-fibronectin, rabbit anti-laminin (all from Sigma-Aldrich) and rabbit anti-collagen IV (Millipore, Massachusetts, USA). Cells were washed and incubated with the appropriated secondary polyclonal antibodies for 1 h at 37 °C. DNA staining was performed with DAPI 0.2 mg/ml, incubated for 5 min at 20 °C and samples were mounted in DABCO/Glycerol solution. For α-SMA, slides were observed in a Nikon Eclipse Ci-E microscope (Nikon). The microscope was coupled to the fluorescence system Intensilight C-HGFI and to the Digital Sight DS-U3 acquisition image system (Nikon). For ECM proteins, slides were analyzed under confocal microscope Zeiss 601 from the Plataforma de Microscopia Óptica de Luz Gustavo de Oliveira Castro (Universidade Federal do Rio de Janeiro, UFRJ). For determination of percentage of SMA-positive cells, fluorescence micrographs were obtained using a 40× objective and a total of 60 microscopic fields per experimental condition (control and *T. cruzi*-infected, 24 hpi) from three independent experiments were analyzed. The number of positive SMA cells was divided by the number of DAPI-positive cells per field and multiplied by 100. Statistical analyses were performed with GraphPad Prism software 5.0 using the Student’s *t*-test. Changes were considered statistically significant when *P* < 0.05.

### Immunoblotting

At desired time-points (6 to 72 hpi), cells were washed with PBS and scraped with 200 μl of RIPA lysis buffer (50 mM Tris-HCl, pH 7.5; 150 mM NaCl; 0.1% SDS; 1% deoxycholate sodium) containing 10% protease inhibitor cocktail (Roche) and phosphatase inhibitor cocktail (Sigma-Aldrich) and samples were frozen at -80 °C until used. Lysates were sonicated and protein concentration was determined using the BCA protein quantification kit (Pierce). Ten micrograms of protein was loaded and resolved in 10% SDS-polyacrylamide gels. The proteins were transferred to nitrocellulose membranes (Bio Rad, Hercules, CA, USA) and incubated with 5% skim milk in TBST (TBS and 0.5% Tween 20) for 30 min followed by incubation with primary rabbit polyclonal anti-fibronectin, anti-laminin and anti-collagen IV antibodies, mouse monoclonal anti-α-SMA and anti-ST antibodies, or goat polyclonal anti-DDR2 antibody diluted in TBST with 5% skim milk overnight at 4 °C. Mouse anti-GAPDH monoclonal antibody was used as loading control. Membranes were washed with TBST followed by incubation with secondary goat anti-rabbit IgG and goat anti-mouse IgG HRP-labeled antibodies for 1 h at 25 °C. Membranes were washed with TBST, incubated with the chemiluminescent kit ECL (Pierce) and exposed to X-Ray film (Thermo Fisher Scientific). The densitometry of bands was performed with the software Image Studio Lite v.4.0. Relative expression of the target proteins (α-SMA, fibronectin, laminin and collagen IV) was determined by the ratio between values of intensity of its band by the values of GAPDH band. Relative expression values from infected samples were normalized by the values of uninfected cultures in the same time point. Statistical analyses were performed with GraphPad Prism software 5.0 using unpaired Student's t-test and one-way ANOVA test with Tukey *post-hoc* test. Changes were considered statistically significant when *P* < 0.05.

## Results

### Establishment of culture enriched in cardiac fibroblast

**Fig. 1:**
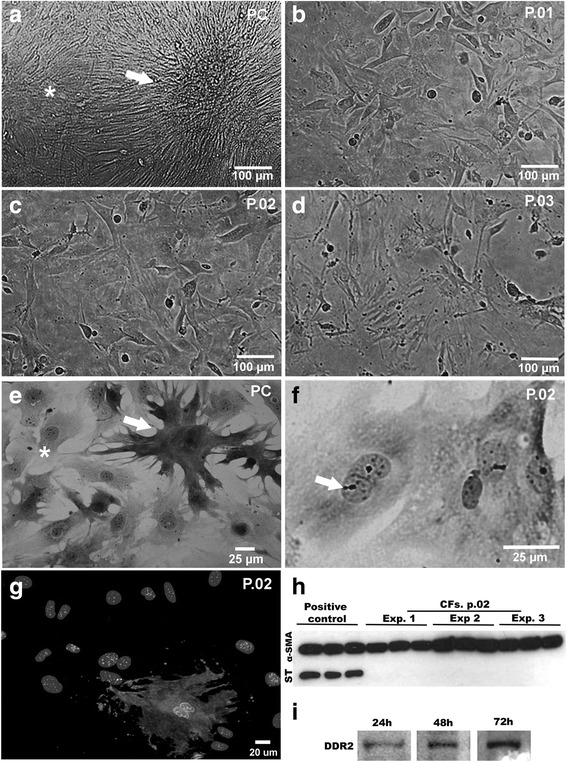
Characterization of culture enriched in cardiac fibroblast. **a**-**f** Phase contrast and bright field microscopy showing features of CFs cultures. **a**, **e** Primary culture presenting cardiomyocytes clusters (arrow) that showed spontaneous contraction, surrounded by CFs (*) forming a monolayer. **b**-**d** Passages 1, 2 and 3, respectively, showing the aspect of CF-enriched cultures. **f** Fibroblast culture at passage 2, stained with Giemsa, demonstrating typical morphology with elongated cells, cytoplasmic extensions, the oval and large nucleus with apparent nucleoli. **g** Immunofluorescence showing sarcomeric tropomyosin expression indicating 95% purity of CFs (nuclei were labeled with DAPI). **h** Immunoblotting for ST revealed that CF cultures were myocyte-free. For positive controls, hearts of mouse embryos were used. Lanes show technical triplicates of three independent experiments (EXP). α-SMA expression, used as load control, can be observed in all samples. **i** Representative immunoblotting demonstrating DDR2 expression in CFs in different times of culture

We established the protocol for the purification of CFs from cardiac myocyte primary cultures based on morphological and biochemical analysis. The cardiomyocyte cultures were subcultured over three passages to define the culture system enriched in CFs (Fig. 1a-d). Primary cultures (72 h) were mainly composed of clusters of contractile cardiomyocytes surrounded by non-contractile sprayed cells as observed by phase-contrast and bright-field microscopy (Fig. 1a, c). After subsequent passage, contractile cardiomyocytes were mostly eliminated and an increase in fibroblast population, forming a confluent monolayer, was noticeable (Fig. 1b, d). Typically, CFs were elongated flattened forms found with a large oval nucleus containing two or more nucleoli (Fig 1f). Based upon morphological aspects, fibroblasts were predominant in second passage cultures.

To confirm the enrichment in CFs, the expression of sarcomeric tropomyosin (ST), a protein specific of striated muscle cells, was evaluated in the second passage cultures (Fig. 1g, h). Immunofluorescence analysis showed that less than 5% of cells were positive for ST, indicating a high enrichment in CFs (Fig. 1g). Additionally, immunoblotting analysis showed the expression of ST only in samples of cardiac tissue (positive control) but it was undetectable in the second passage of cardiac cell cultures (Fig. 1h). Although no general marker for cardiac fibroblasts has been identified to date, a tyrosine kinase receptor named discoidin domain receptor 2 (DDR2), which is activated by collagen, is specifically expressed by CFs but not by the other cell types [20]. The expression of DDR-2 was evaluated during the establishment of CF cultures and was found to be expressed in all time points analyzed (24, 48 and 72 h) (Fig. 1i).

### *Trypanosoma cruzi* successfully completes its intracellular cycle in enriched cardiac fibroblasts cultures

**Fig. 2:**
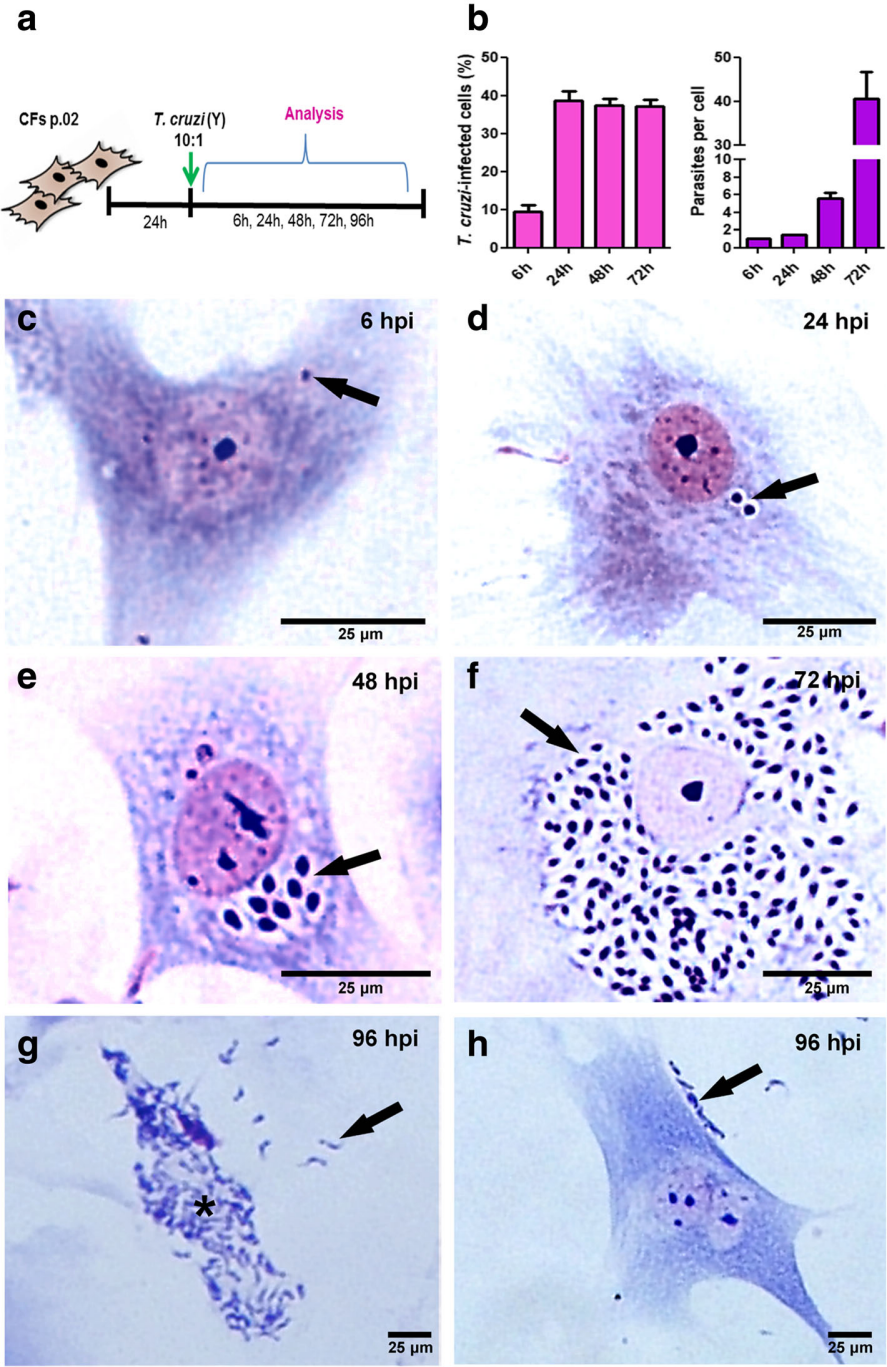
*Trypanosoma cruzi* intracellular cycle in cardiac fibroblasts. **a** Schematics of the experimental design: fibroblast cultures were infected at passage 2 and analyzed after 6 to 96 h of infection (MOI 10). **b** Quantitative data of the infection of CF by *T. cruzi*. At 6 hpi 9.4% of the host cells are infected by one parasite and from 24 to 72 hpi the infectivity was of 38%. Proliferation of the intracellular amastigotes started at 48 hpi with 5 parasites/infected cell and reached 40 parasites/infected cell at 72 hpi. **c**-**h** Bright field microscopy representative images of *T. cruzi* infected CFs cultures stained with Giemsa showing the parasite intracellular cycle. In **c** intracellular parasites are visible already after 6 h of infection (arrow). **d** At 24 h post-infection we can observe the beginning of the amastigotes proliferation process (arrow). **e** At 48 h an increased number of intracellular amastigotes can be observed. **f** After 72 h of infection a high number of amastigote forms are seen through all cytoplasm. **g** At 96 h of infection is possible to see that the parasites differentiated to trypomastigotes (asterisk) and evaded the host cells (arrow). **h** After evading host cells, the released parasites attach to new CFs to restart a new cycle (arrow)

Our next step was to establish the infection of CFs cultures by *T. cruzi*. After 24 h of plating, CFs were infected by trypomastigote forms of *T. cruzi* (Y strain) using a MOI 10 and the infection was maintained during 6, 24, 48, 72 and 96 h (Fig. 2a). Giemsa staining revealed that *T. cruzi* infected the CFs and completed their intracellular cycle (Fig. 2b-g) and quantitative analyses revealed that at 6 hpi, 10% of the culture was parasitized, with an average of 1 parasite per infected cell. At 24 hpi the infectivity rate was 38% of the cells and this percentage was maintained until 72 hpi (Fig. 2b). The parasites invaded the CFs (Fig. 2c), differentiated into amastigote forms (Fig. 2d), proliferated (Fig. 2e, f) and reached an average of 40 intracellular parasites per infected cell. Then amastigotes differentiated into trypomastigotes and evaded the host cells (Fig. 2 g) and finally, adhered in new cells to restart a new intracellular cycle (Fig. 2h). These data confirm previous results obtained in primary cultures of cardiac cells presenting CFs but enriched in cardiomyocytes [19].

### *Trypanosoma cruzi* induces fibroblast-myofibroblast transition

**Fig. 3:**
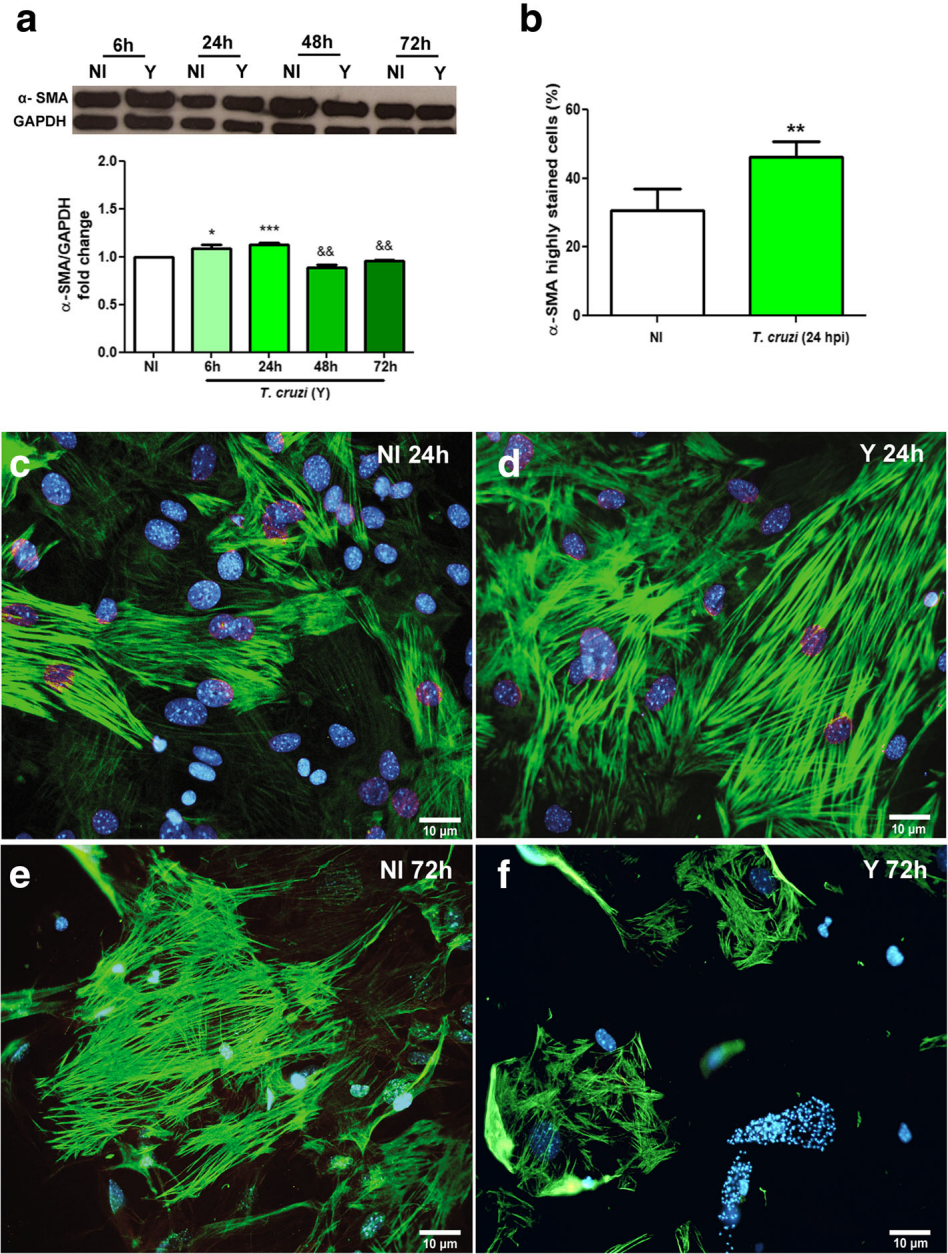
Fibroblast-myofibroblast transition induced by *T. cruzi* infection. **a** Immunoblotting was performed to evaluate α-SMA expression in CFs cultures. α-SMA expression is increased at 6 h and 24 h of *T. cruzi* infection, followed by a decrease at 48 h and 72 h of infection. GAPDH was used as loading control. **b** Quantitative analysis of the percentage of SMA-stained cells in CF cultures at 24 hpi. Uninfected cultures showed an average of 30% positivity, whereas infection led to a 1.5-fold increase in the number of stained cells. **c**-**f** Immunofluorescence revealed the labelling pattern of α-SMA filaments in CFs cultures. **c**, **e** Uninfected cultures. **d**, **f** *T. cruzi*-infected cultures. **d** At 24 hpi it is possible to observe an increase in α-SMA signal in comparison to non-infected control (**c**). **f** At 72 hpi, α-SMA immunoreactivity was drastically altered when compared to non-infected controls (**e**)**.** DNA staining with DAPI can be observed in blue. Values are expressed as fold change of infected cultures by their respective controls (NI) ± SEM. *P < 0.05 (stimulus); && *P* < 0.01 (inhibition); ***P < 0.001 (stimulus). One-way ANOVA with Tukey’s *post-hoc* test of three independent experiments. *Abbreviations*: NI, non-infected cultures; Y, *T. cruzi*-infected cultures

Since the activation of myofibroblasts is the initial stage on the fibrogenesis in injured tissues [10], we questioned whether *T. cruzi* infection would activate the differentiation of CFs into myofibroblasts. This differentiation event was analyzed by assessing changes in α-SMA expression, a cytoskeleton protein that is highly expressed in activated myofibroblasts [13]. By western blot, our results demonstrated a 9% increase in the expression of α-SMA was observed after 6 h of infection (hpi) (*t* = 3.320, *df* = 6, *P* = 0.0160), achieving its maximum at 24 hpi (12%) (*t* = 7.072, *df* = 7, *P* = 0.0002). Later times of infection led to a decrease in α-SMA levels, showing 11% (*t* = 4.701, *df* = 7, *P* = 0.0022) and 4% (*t* = 4.783, *df* = 7, *P* = 0.0020) less expression at 48 and 72 hpi, respectively (Fig. 3a). By immunofluorescence we verified that *T. cruzi* infection led to an increase of 50% in the percentage of SMA-positive cells at 24 hpi (*t* = 4.578, *df* = 8, *P* = 0.0018) (Fig. 3b). The distribution of α-SMA was also evaluated in CFs after 24 and 72 h of *T. cruzi* infection (Fig. 3c-f). Early in the infection (24 hpi), fibroblasts showed a strong immunoreactivity for α-SMA without alterations on the pattern of distribution of the protein filaments (Fig. 3c, d), indicating the activation of quiescent cardiac fibroblasts. In contrast, at a later time point (72 hpi), a slight reduction of α-SMA fluorescent signal was observed in non-infected or low infected cells and also a disarray of the α-SMA filaments in highly infected cells (Fig. 3e, f).

### Infected CFs produce higher levels of extracellular matrix proteins

**Fig. 4:**
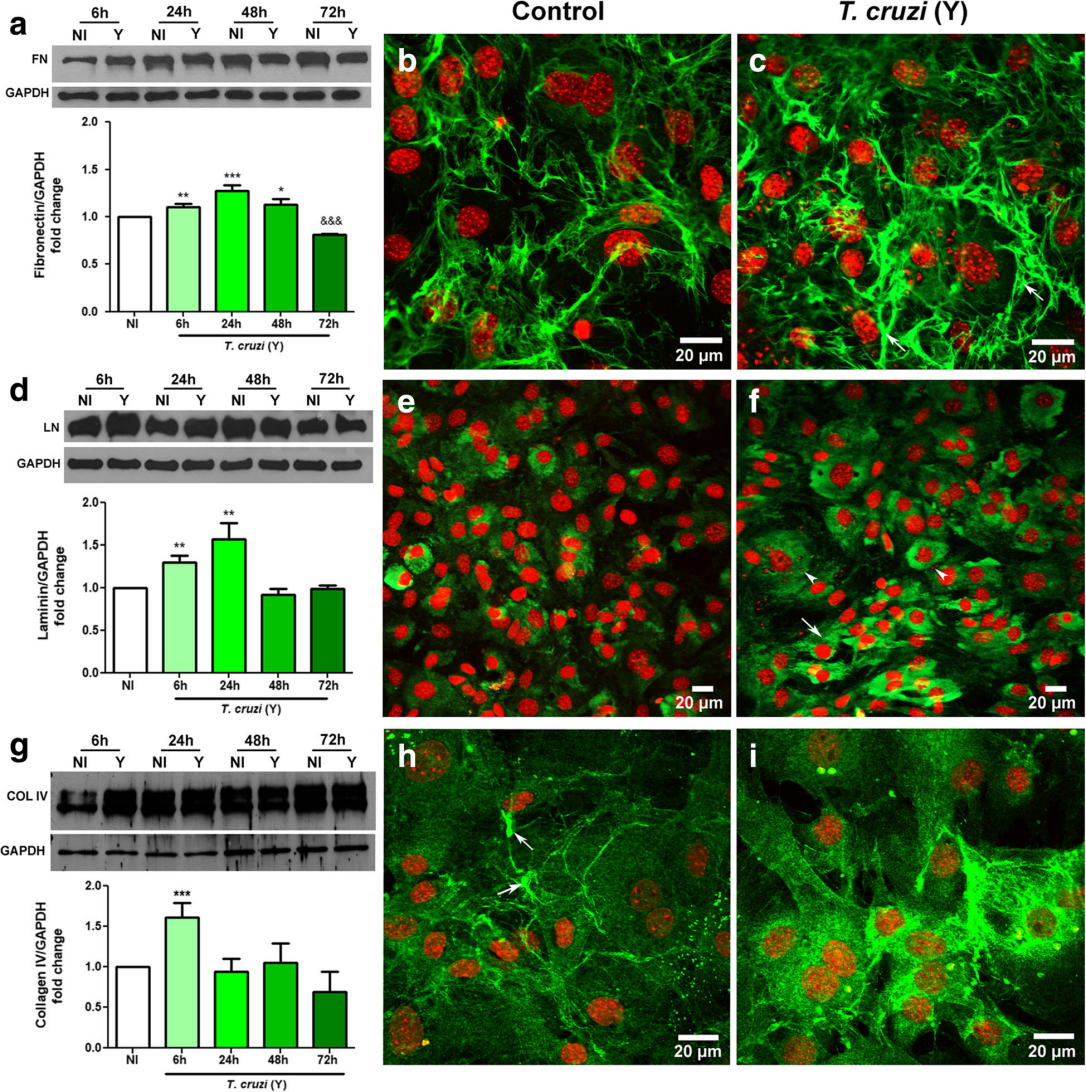
*Trypanosoma cruzi* infection alters ECM components expression by CFs. **a**, **d**, **g** Immunoblotting performed to ECM proteins in CFs cultures and their densitometric analyses plotted as graphs. **a-c** Fibronectin (FN) expression was increased after 6, 24 and 48 h of *T. cruzi* infection, followed by a decrease at 72 hpi. Immunofluorescence for FN (green) showed that at 24 hpi *T. cruzi*-infected cultures (**c**) had higher deposition of this protein (arrows) when compared to uninfected dishes (**b**). **d-f** Laminin (LN) is increased after 6 and 24 h of infection as shown by immunoblot. **e** and **f** show immunolocalization of LN in CF. In non-infected cultures LN showed a cytoplasmic distribution (in green). At 24 hpi, infected cultures displayed patches of cells with a more intense signal for LN (arrows), that corresponded with parasitized cells (arrowheads for intracellular amastigotes). **g-i** Collagen IV (COL) is transiently increased by *T. cruzi* infection. Immunoblot for COL showed a 1.5-fold increase at 6 hpi compared to uninfected cultures (**g**). COL had a fibrillar pattern in uninfected cultures (arrows in **h**) while in *T. cruzi* infection (6 hpi), COL immunoreactivity was increased. GAPDH was used as loading control. DAPI stained the DNA of host cells and of intracellular parasites (arrowheads) and is shown in red (pseudo-color). Values are expressed as fold change of means of the analyzed samples by means of their respective controls (NI) ± SEM. Representative data from three independent experiments. *P < 0.05 (stimulus), **P < 0.01 (stimulus), ***P < 0.001 (stimulus), &&& *P* < 0.001 (inhibition). One-way ANOVA with Tukey’s *post-hoc* test. *Abbreviations*: NI, non-infected cultures; Y, *T. cruzi*-infected cultures. *Scale-bars*: 20 μm

An important aspect of myofibroblasts activation is the production and deposition of extracellular matrix proteins [16, 18]. For this reason, we assessed whether the infection by *T. cruzi* exacerbates the production of ECM proteins by CFs. Our results demonstrated an increase in the protein contents of fibronectin (FN), laminin (LN) and collagen IV (COL) after *T. cruzi* infection (Fig. 4a-d). The enhancement of these proteins’ content was clearly associated with myofibroblast differentiation. FN was significantly increased at 6 (11%) (*t* = 4.131, *df* = 6, *P* = 0.0061), 24 (27%) (*t* = 5.570, *df* = 7, *P* = 0.0008) and 48 (13%) hpi (*t* = 2.962, *df* = 6, *P* = 0.0252) (Fig. 4a). Confocal microscopy confirmed that FN deposition was increased at 24 hpi, with denser fibers between the cells (Fig. 4b, c). An overexpression of LN was also observed in the early stages of infection (6 and 24 h), reaching 30% (*t* = 5.01, *df* = 6, *P* = 0.0024) and 56% (*t* = 4.059, *df* = 6, *P* = 0.0067) higher signal than uninfected controls, respectively (Fig. 4d). By immunofluorescence, heterogeneous patterns of laminin staining was observed in *T. cruzi*-infected cultures, with different degrees of intensity that did not correlate with the proximity of a parasitized cell (Fig. 4e, f). COL had its content transiently increased by the infection, since western blot data showed that at 6 hpi infected cultures had 60% more collagen IV than uninfected controls (*F*_(4,16)_ = 13.96, *P* < 0.0001) (Fig. 4g). After this time-point, COL levels in infected cultures were restored to what was observed in the controls. This was evident in by confocal microscopy, since COL immunoreactivity was deeply increased in infected cultures, when compared to the non-infected cultures (Fig. 4h, i).

## Discussion

In this study, we investigated the effect of *T. cruzi* on the activation of cardiac fibroblasts in vitro, trying to understand the contribution of these cells in cardiac fibrosis during Chagas disease. We observed that *T. cruzi* infection activated CFs, inducing differentiation to myofibroblasts and increasing ECM proteins synthesis.

Crucially, fibroblasts take part in the genesis and maintenance of fibrosis during injury situations, when the tissue repair machinery is necessary [16]. The involvement of CFs in fibrosis and tissue remodeling in cardiovascular diseases such as myocardial infarct, hypertension and heart failure, has been described [12, 14, 17]. Moreover, cardiac fibrosis causes electrical alterations and heart failure [11, 13, 21]. The persistence of pro-fibrotic stimulation then results in an activated phenotype of fibroblasts and, consequently, an increase in the production and accumulation of ECM proteins [16].

The cardiac fibrosis is an important manifestation of Chagas disease [3] and the role of CFs in this process is understudied. However, the association of CFs with inflammatory cells, myofibroblasts and collagen deposits was demonstrated in an experimental model of CCC [6], suggesting a possible participation CFs on heart tissue remodeling during *T. cruzi* infection.

Our first step in this study was to settle and characterize cell cultures enriched in CFs. Such enrichment was confirmed by the absence of sarcomeric tropomyosin expression, a protein of striated muscle cells including cardiomyocytes [22] and by the presence of DDR-2, a collagen receptor expressed exclusively in CFs [20]. Moreover, the CFs in culture preserved the morphological and functional characteristics as previously observed in vivo [23] and in vitro [20, 24]. These cells were capable of differentiating into myofibroblasts, as revealed by the expression of α-SMA and were able to produce ECM proteins, including laminin, fibronectin and collagen IV, similarly to what is observed in vivo [13, 16]. Indeed, in an experimental model of chronic Chagas disease in mice, the expression of α-SMA was increased in the heart tissue [25].

The establishment of *T. cruzi* infection in CFs-enriched cultures was based on previous studies of our group approaching aspects of the interaction of *T. cruzi* with cardiac cells [17]. Herein, we observed that trypomastigote forms of *T. cruzi* successfully invaded CFs. Furthermore, the parasite completed its intracellular cycle inside CFs. These data are in agreement with previous data of our group supporting the infection and intracellular cycle of *T. cruzi* parasites in CFs of primary cultures of cardiac cells enriched in cardiomyocytes [26]. Interestingly, using this mixed culture of cardiac cells [27], a group described that infected CFs were refractory to infection-induced apoptosis, indicating that these cells may be activated in such an inflammatory environment.

Our data showed that *T. cruzi* infection led to a transient increase in expression of SMA, thus indicating fibroblast-to-myofibroblast transition. Although the overall increase in SMA content was of only 9–12%, our immunofluorescence analyses showed that at the same time point (24 hpi) the number of highly stained cells for SMA was higher than in uninfected controls. However, when a protein lysate is generated for western blotting, this effect might be diluted. Additionally, at the final times of infection, as the parasite replicates in the cytoplasm, there was a reduction in α-SMA expression and disorganization of this protein, suggesting a breakdown of the cytoskeleton, as previously shown in cardiomyocytes [28, 29]. However, paracrine effects of secreted factor by infected CFs could maintain the culture in an activated state. Previous studies using mixed cultures of mouse and human cardiac cells have shown that *T. cruzi* induce the activation of TGF-beta [30] and pro-fibrotic proteins [31] as well as inflammatory cytokines [32].

*Trypanosoma cruzi* secrete soluble factors that may also induce production of ECM proteins in fibroblast cultures [33]. Moreover, cytokines produced by infected cells may contribute to fibroblast-myofibroblast transition. The consequence of myofibroblast differentiation is the increased expression and deposition of ECM proteins that further contribute to the fibrotic process [16]. Inflammation and fibrosis are hallmarks of CCC [6, 34–36], with production of cytokines that contribute to pathogenesis [37] and co-localization of *T. cruzi* DNA or antigens with inflammatory infiltrate and fibrosis in cardiac tissue was also observed [6, 7, 38].

We were interested in verifying whether in our system of purified CFs *T. cruzi* would stimulate ECM (fibronectin, laminin and collagen IV) deposition as seen in the cardiac tissue during in vivo infection [7, 39–42]. In the present study, we showed that *T. cruzi* infection, without the presence of inflammatory cells, increased the production of both fibronectin and laminin at 6 and 24 hpi, being reduced at the later time points. Although in the chagasic heart the levels of ECM proteins is extremely higher compared with what we observed in our CF cultures, the fact that fibronectin needs a three-dimensional microenvironment to establish a firbillar network [43] must be taken into consideration when analyzing data from monolayer cultures. This transient increase in ECM proteins in infected CF differs from what was shown in mixed cultures of cardiac cells [39]. Cytoskeleton breakdown can also be responsible for ECM decrease in heavily infected cells in this 2D system, since previous work done on mixed cultures of cardiac cells showed that these two proteins are decreased at late stages of infection [39]. Increased production of ECM proteins by *T. cruzi* infection or *T. cruzi* secreted factors in skeletal muscle cells (C2C12) and adipose tissue fibroblasts (L929) have also been demonstrated in the early stages of infection/treatment [44]. In addition, our group had previously shown that *T. cruzi* induces fibrosis, hypertrophy and changed the distribution pattern of ECM proteins in a three-dimensional (3D) cardiac cells culture system [8].

ECM deposition observed in fibrosis leads to tissue stiffness, which contributes to cardiac electrical alterations [45]. In fact, electrocardiographic (ECG) abnormalities are found in chronically infected mice and humans, concomitantly with the presence of fibronectin increase and myocardial scars that correlate with a worse prognosis in chronic patients [42, 46].

In conclusion, our study showed that *T. cruzi* is able to activate CFs, possibly contributing to cardiac fibrosis in Chagas disease. Therefore, the utilization of therapeutic strategies including trypanocidal compounds could contribute to inhibit the activation of CFs, reducing the synthesis of ECM proteins and consequently the fibrosis, improving the prognosis of patients with chronic Chagas cardiomyopathy.

## Conclusions

Based on our results we conclude that (i) the culture of purified cardiac fibroblasts will serve as an important tool to deepen the knowledge of the pathogenesis of chagasic cardiomyopathy and (ii) *T. cruzi* infection activates these cells, which in turn contributes further to ECM deposition in response to infection. In the context of the in vivo infection we believe that cardiac fibroblasts may serve as an important cellular target to prevent exacerbation of tissue fibrosis.
